# Exercise in type 1 diabetes: real-world data on glucose levels and hypoglycaemia risk from over 420,000 exercise sessions

**DOI:** 10.1007/s00125-026-06672-y

**Published:** 2026-02-13

**Authors:** Josip Zivkovic, Michael Mitter, Delphine Theodorou, Othmar Moser, Timor Glatzer

**Affiliations:** 1 Data, Analytics & Research; mySugr GmbH, Vienna, Austria; 2 Data, Analytics & Research; Roche Diagnostics International, Basel Branch of Diabetes Care, Basel, Switzerland; 3 Exercise Physiology, Training and Training Therapy Research Group, Training and Diagnostic Centre, Department of Human Movement Sciences, Sport, and Health, University of Graz, Graz, Austria; 4 Interdisciplinary Metabolic Medicine Trials Unit, Division of Endocrinology and Diabetology, Medical University of Graz, Graz, Austria; 5 Clinical Development, Roche Diabetes Care GmbH, Mannheim, Germany

**Keywords:** Aerobic exercise, Anaerobic exercise, Exercise timing, Exercise-induced glycaemic effects, Glucose management, Glycaemic management, Hypoglycaemia risk, Nocturnal hypoglycaemia, Walking

## Abstract

**Aims/hypothesis:**

This large observational cohort real-world study explored the effects of three forms of exercise (walking [WALK], aerobic excluding walking [AER] and anaerobic [ANAER]) on glucose levels and hypoglycaemia risk in type 1 diabetes.

**Methods:**

Data were collected from 3248 users of mySugr Logbook and Apple Health (mean ± SD age 41.23±12.25 years; glucose management index of 7.05±1.09%; 41.5% were female) over a total of 428,058 exercise sessions. Acute and 24 h glycaemic effects were examined across exercise types. Post-exercise glycaemia data over 24 h were compared with sedentary glycaemic data. Time of exercise was used to assess the probability of nocturnal hypoglycaemia.

**Results:**

Independent of type, exercise decreased glucose by −1.06±0.89 mmol/l. For the individual types of exercise, WALK decreased levels by −1.24±0.81 mmol/l, AER by −1.43±1.02 mmol/l and ANAER by −0.52±0.81 mmol/l (all *p*<0.001). Comparing sedentary days vs active days, the time in range (3.9–10 mmol/l glucose) increased by +2.08±6.06% for WALK, +2.94±6.46% for AER and +3.93±7.16% for ANAER, and the time below range (<3.9 mmol/l) increased by 0.37±1.57% for WALK, 0.74±1.70% for AER and 0.68±1.79% for ANAER (all *p*<0.001). ANAER yielded a smaller chance of acute hypoglycaemia and WALK yielded a smaller chance of nocturnal hypoglycaemia (*p*<0.001). Activities done after 15:30 hours did not increase the risk of nocturnal hypoglycaemia when compared with earlier exercise sessions (+0.9±0.34%; *p*<0.01).

**Conclusions/interpretation:**

Aerobic activities decreased glucose more during exercise sessions than anaerobic exercise and yielded larger acute hypoglycaemia risk; anaerobic activities yielded the largest 24 h glycaemic improvements. More-intense exercise resulted in a larger nocturnal hypoglycaemia than walking; exercise timing was not a relevant contributor to nocturnal hypoglycaemia.

**Graphical Abstract:**

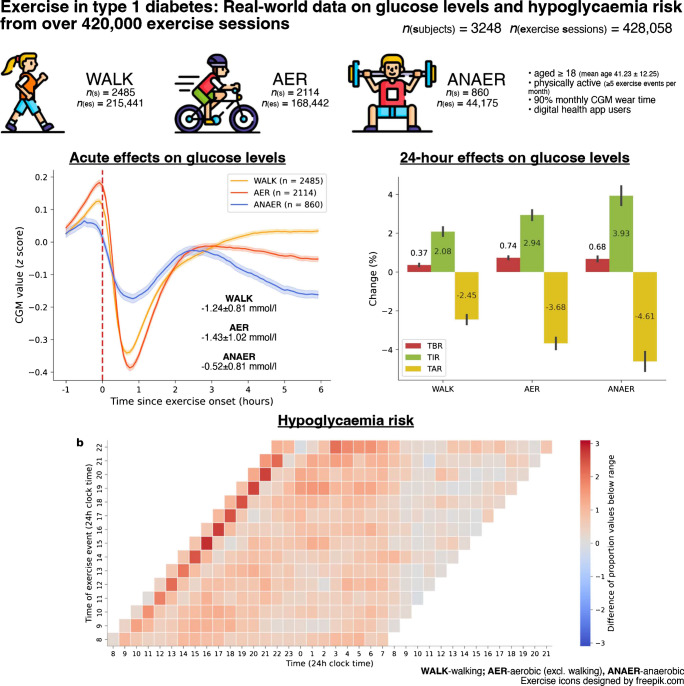

**Supplementary Information:**

The online version contains peer-reviewed but unedited supplementary material available at 10.1007/s00125-026-06672-y.


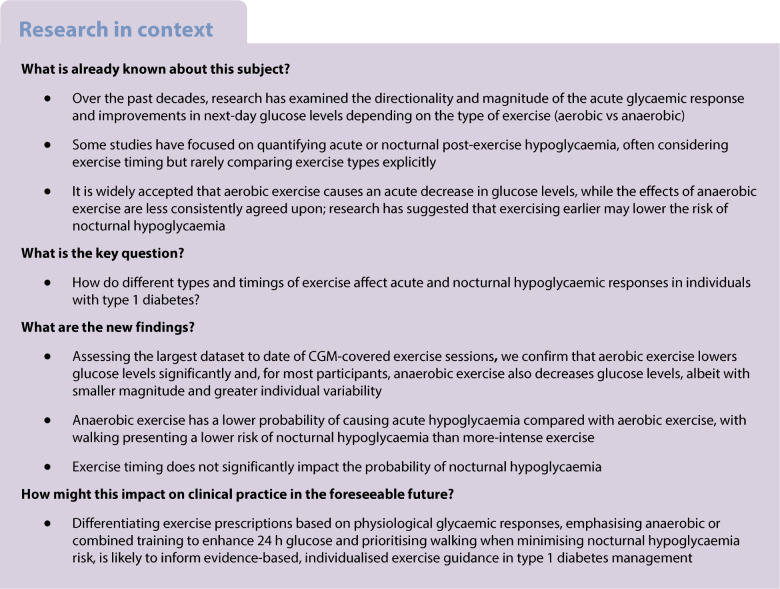


## Introduction

Physical activity is highly recommended for individuals with type 1 diabetes [1], for its improvements in glycaemic management [2, 3], improved cardiovascular fitness [4], decreased insulin requirements, improved body composition and enhanced quality of life [5, 6]. The effects of different exercise types on acute and extended glycaemia in individuals with type 1 diabetes are widely discussed in the literature [7]. It is generally accepted that in the short-term, aerobic forms of exercise generally acutely decrease glucose levels, whereas the directionality and the magnitude of the effect of anaerobic forms of exercise are more debated [6, 8–10].

The increased risk of exercise-induced hypoglycaemia is a major barrier for incorporating physical activity into the daily routines of those administering insulin, particularly among people with type 1 diabetes [1, 11]. Research has shown that post-exercise hypoglycaemia risk can be biphasic [12], presenting immediately (during/after exercise) and as a delayed, often nocturnal risk [13, 14]. Clinical guidelines advise proactive caution for both acute and nocturnal post-exercise hypoglycaemia [1, 15] to mitigate exercise-induced dysglycaemia. Additionally, it has been suggested that the time of day when exercise is performed plays a role in mitigating nocturnal hypoglycaemia risk, with earlier exercise potentially resulting in a lower risk of subsequent nocturnal episodes when compared with late afternoon/evening exercise [13, 16]. However, the exact relationship between the timing of exercise, type of exercise and subsequent risks of hypoglycaemia in the 24 h period following exercise remains insufficiently explored.

In recent years, the technological evolution of continuous glucose monitoring (CGM), as well as wearable activity-tracking sensors, has enabled a new way to gather insights from real-world data (RWD). The combined quality of high-resolution glycaemic data with exercise-related information from wearable sensors offers a new way to collect large amounts of data outside of controlled laboratory settings. This integration of diverse data sources allows us to analyse relationships between glucose levels and physical activity in daily life, resulting in more comprehensive and practical insights. RWD analyses have already supported the notion that exercise improves glycaemic management in people with type 1 diabetes, with a study from the T1DEXI-P initiative exploring short-term changes in glycaemia after exercise in adults and adolescents with type 1 diabetes [17], two studies quantifying daily improvements in glycaemic management between exercise and no-exercise days in adult population [2, 18], and one study exploring the risk of immediate post (aerobic)-exercise hypoglycaemia [19]. Research from the T1DEXI study has also shown that exercise is an important contributor to nocturnal hypoglycaemia [18, 20].

To date, studies have not used a single data source to examine the differences in glycaemic patterns and (nocturnal) hypoglycaemia risk within 24 h post-exercise depending on the type and timing of exercise. While exercise days are seemingly associated with a higher risk of subsequent nocturnal hypoglycaemia compared with sedentary days, the precise influence of exercise timing (e.g. late afternoon vs morning) on this risk remains a subject of ongoing debate across large cohort studies. This long-recognised clinical problem, established in early diabetes literature, continues to require clarification regarding the time-of-day effects on post-exercise nocturnal hypoglycaemia. Hence, in this study, we used real-world glycaemic data from CGM devices and exercise data from Apple Health, gathered from type 1 diabetes users of the mySugr app [21]. This comprehensive dataset allowed us to investigate the interplay between exercise and overall glycaemia and hypoglycaemia risk.

Overall, we aimed to contribute to a better understanding of how exercise modulates glycaemia and how it contributes to both diurnal and nocturnal hypoglycaemia. Remedying exercise-induced hypoglycaemia risk can support people with type 1 diabetes in conducting exercise more safely, thus harvesting the beneficial effects on glycaemia while minimising the associated risk of hypoglycaemia-induced adverse events.

## Methods

### Data source and data collection

Exercise and CGM data from mySugr Logbook and Apple Health were obtained from 3248 mySugr users with type 1 diabetes who provided electronic consent for data processing, covering an observation period from March 2015 to January 2025. The mySugr Logbook, a Class II medical device in Europe, is a supportive diabetes management tool that integrates CGM- and diabetes-relevant information from Apple Health, providing users with a platform for a holistic approach to diabetes monitoring.

### User cohort definition

Data were included from 3248 users with a minimum 90% monthly CGM coverage for each included user-month. The sample was further defined by requiring users to have at least five valid exercise events during their CGM wear-time and a minimum of 2 weeks of sedentary data. As some users had more than five events in each exercise category (walking [WALK], aerobic, excluding walking [AER], and anaerobic [ANAER]), the sum of the final subsamples per type (5459; comprising WALK 2485; AER 2114; ANAER 860) was larger than the total of 3248 users. Demographic information about sex and age was self-reported.

### Ethical considerations

All app users provided consent to their data being used for research purposes, which covers their use within the context of this publication. In addition, we submitted the research protocol for this publication to an independent institutional review board from WCG (https://www.wcgclinical.com/about/), which reviewed and determined it to be exempt under [45 CFR 46.104(d)(4)]. The exemption applies to secondary research using identifiable private information or biospecimens that meet specific regulatory criteria.

### Event-mapping and event characteristics

Similarly to the protocol in the T1DEXI study [18], valid exercise sessions (428,058) were ensured to last a minimum of 15 min to guarantee sufficient length to potentially impact glycaemia. A maximum duration threshold (3 SDs from the mean, or the 99th percentile, after the 15 min minimum filter) was applied per exercise type to exclude very long events likely logged incorrectly. All exercise sessions were tagged and imported by Apple Health, and available tags were used to classify fitting events into one of the three categories (Table 1).

**Table 1:** Descriptive statistics of the events included in the analysis, stratified by exercise type

Exercise type	No. of exercise sessions	Duration (min)	Minimum duration (min)	Median duration (min)	Maximum duration (min)	No. of unique users
WALK	215,441	41.33±24.38	15	34.16 (23.60–51.6)	158.15	2485
AER	168,442	48.64±35.72	15	37.06 (25.91–59.53)	245.93	2114
Cycling	93,880	52.43±26.39	15	38.53 (25.01–62.8)	245.93	1642
Running	45,382	41.68±20.03	15	36.58 (27.80–51.67)	135.52	1337
Other aerobic	29,180	47.30±35.07	15	35.35 (25.30–57.15)	221.92	1269
ANAER	44,175	49.61±26.39	15	45.25 (30.35–62.46)	233.85	860

Data are presented as mean ± SD or median (IQR)

Event types, excluding walking, were categorised into AER or ANAER groups based on their expected physiological mechanisms [10]. Apple Health activities tagged as cycling, running, cross-training, swimming, rowing, hiking, stair climbing, elliptical, mixed cardio and cardio dance were grouped under AER; activities tagged as traditional strength training, functional strength training, gymnastics and core training were grouped under ANAER. Walking was assessed independently due to its commonality and lower intensity compared with other aerobic activities.

### Metric calculation and statistical analyses

CGM data were resampled to 5 min intervals for all available user information, and CGM values were aggregated to the mean in each 5 min bin. Observation windows for exercise were defined from exercise onset to 24 h after the logged exercise event for the purposes of metric calculations, and from −2 h to +24 h for visualisation. Based on the 2023 T1DEXI study protocol [18], all unique user-level CGM timestamps in the 24 h post-exercise window were defined as ‘post-exercise data’, while the opposite set of timestamps constituted the ‘sedentary days’. Invalid events, excluded due to unfitting exercise type or duration, were accounted for and excluded from sedentary data.

#### Immediate post-exercise glycaemic metrics

To visualise individualised glycaemic variability around exercise onset, CGM values were standardised to *z* scores within each user-month and then averaged around the exercise time per exercise type. This standardisation allowed more precise comparisons across individuals and exercise types by accounting for personal glycaemic baselines and variability.

To assess glucose changes around exercise times, the CGM value change from exercise onset to end was calculated for each session. The change values were averaged within user and exercise type and compared across exercise types using a one-way ANOVA test and subsequent Tukey’s honestly significant difference (HSD) tests for pairwise comparisons.

Finally, to quantify 1 min glucose changes per exercise type, change values from exercise onset to the end were divided by session duration. This rate of change was averaged within user and exercise type, and compared across exercise types using a one-way ANOVA test and subsequent Tukey’s HSD tests for pairwise comparisons.

#### 24 h post-exercise glycaemic metrics

To assess exercise's extended impact on glycaemia, 24 h post-exercise glycaemic values were examined and contrasted with users' sedentary-day data.

#### 24 h post-exercise glucose response

Mean post-exercise CGM values for the 24 h following exercise end were calculated per user and exercise type and compared with the mean sedentary CGM value for each user-month. This allowed calculation of a glycaemic decrease value, estimating exercise-induced glucose reduction relative to sedentary levels over 24 h. Specifically, the difference between CGM values 24 h after exercise and the user’s mean sedentary value was computed for each exercise event, and averaged to obtain the mean post-exercise difference from the sedentary mean. These mean differences were then averaged within users and exercise types to determine the mean glucose decrease 24 h after exercise across types, and compared across exercise types using a one-way ANOVA on mean decreases, followed by Tukey’s HSD tests for post hoc comparisons.

#### Change of proportions of time in range

Time in range (TIR) (3.9–10 mmol/l glucose), time below range (TBR) (<3.9 mmol/l glucose) and time above range (TAR) (>10 mmol/l glucose) were defined as percentage proportions of CGM values in the respective categories, following Ambulatory Glucose Profile (AGP) guidelines [22, 23]. Proportions in each category were calculated in the 24 h after exercise and contrasted with the corresponding sedentary data proportions for each user. One-way ANOVA was conducted for each glycaemic category across exercise types, with Tukey’s HSD tests used for pairwise comparisons of differences in proportions.

#### Hypoglycaemia probability metrics

To study the interaction between exercise onset time and 24 h hypoglycaemia risk, mean hourly proportions of CGM values <3.9 mmol/l post-exercise were visualised as a function of exercise onset hour. These were contrasted with hourly proportions from sedentary days to assess exercise's effect on hourly hypoglycaemia from baseline. The exercise onset time-window was limited to 08:00 hours to 23:00 hours due to scarce event data outside these times.

To assess whether different exercise types yielded different hypoglycaemia risk patterns over time, acute (2 h after exercise onset) and nocturnal (00:00 hours to 06:00 hours on the night after exercise) post-exercise risks of hypoglycaemic episodes were compared across types. A hypoglycaemic episode was defined as four consecutive CGM readings (15 min) or more, below 3.9 mmol/l, as defined by Eichenlaub et al [24]. Occurrences of such episodes were compared in the acute and nocturnal time bins across exercise types. Additionally, post-exercise hypoglycaemia probabilities were contrasted against baseline hypoglycaemia probabilities from sedentary data. Sedentary nocturnal baseline was derived from the proportion of nights in the sedentary data during which a user experienced a hypoglycaemic event. Sedentary diurnal (06:00 hours to 00:00 hours) baseline was calculated by obtaining the proportion of days with diurnal hypoglycaemia. To match the length of the 2 h acute observation window after exercise, this probability was divided by 9 (corresponding to nine sets of 2 h periods between 06:00 hours and 00:00 hours) to obtain the baseline probability of a hypoglycaemic event in any 2 h diurnal period. These baseline probabilities were subtracted from corresponding post-exercise probabilities to estimate exercise's contribution.

Absolute (uncorrected) and adjusted (baseline-corrected) probabilities of acute and nocturnal hypoglycaemia were compared across exercise types using one-way ANOVA tests, with subsequent Tukey’s HSD tests for pairwise comparisons. Bonferroni-corrected paired-samples *t* tests were used to assess whether absolute and adjusted hypoglycaemia probabilities differed between acute and nocturnal times within each exercise type.

To assess the influence of exercise timing on the probability of nocturnal hypoglycaemia, the available exercise onset time-window (limited to 08:00 hours to 23:00 hours due to scarce event data outside these times) was divided into two time bins: early (before 15:30 hours); and late (after 15:30 hours). We opted for this dual split, using 15:30 hours as the midpoint of the observation window, to obtain matched subsamples of users who had sufficient exercise events logged in both periods for each exercise type. This simplified division was implemented to avoid further dilution of subsamples, which would have occurred if splitting into three or more time bins, and reduced the need for additional formal statistical comparisons and Bonferroni corrections. Matched subsamples were analysed to assess whether later exercise contributed to a larger probability of nocturnal hypoglycaemia. These included 1895 (WALK), 1509 (AER) and 491 (ANAER) users who had three or more exercises logged in both early and late time bins. Proportions of nocturnal hypoglycaemia following exercise (defined as an event occurring between 00:00 hours and 06:00 hours on the night following exercise) were then compared between the two time bins using paired-samples *t* tests.

We hypothesised that aerobic forms of exercise would cause larger acute changes in glycaemia than anaerobic forms. Further, we hypothesised that 24 h changes in glycaemia would also differ across exercise types, and that later exercise would induce a larger hypoglycaemia risk than exercise done earlier in the day.

Statistical significance was determined using *p*<0.01.

## Results

### User- and event sample characteristics

Mean exercise duration independent of the type of exercise was 45.05±29.76 min, with different exercise types noted to have different durations (one-way ANOVA for mean event duration across exercise types *F*=14.45, *p*<0.001; Table 1).

Sex data were available for 63.3% of participants, whereby 58.5% were male. A minimum age of 18 years was used as an inclusion criterion, yielding a mean ± SD age of 41.23±12.25 years and diabetes duration of 19.13 ± 13.24 years. Insulin injection modality was reported as pen in 62.87% and pump in 35.99% of the sample, with 1.14% of the sample not providing information. Users were from a global sample, with 30% coming from the USA, 24% from Germany, 12% from the UK and the rest from an additional 65 countries. BMI data were available for 591 users, who in all months of their CGM use had a mean ± SD BMI of 26.3±5.4 kg/m^2^.

Users had a median (IQR) of 40,350 (20,439–86,438) CGM timestamps in their sedentary data and 11,588 (4051–32,821) in their exercise data, corresponding to approximately 140 (72–300) and 40 (14–114) days, respectively.

### Exercise onset and acute post-exercise glycaemia

**Fig. 1:**
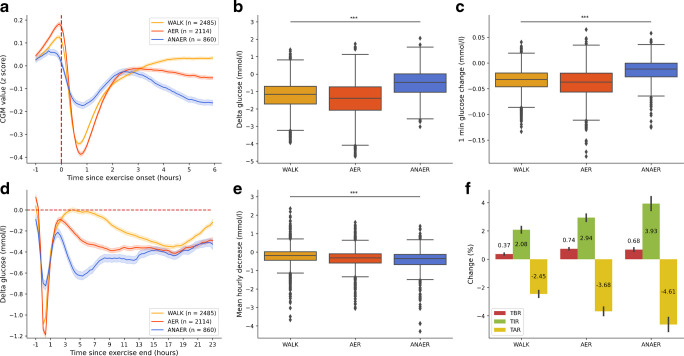
Overall effects of exercise immediately after and 24 h after exercise. (**a**) Mean standardised CGM metrics before and shortly after exercise onset, stratified by exercise type. (**b**) Distribution of change values from exercise onset to end of exercise across exercise types. (**c**) Distribution of rate of change in glucose per 1 min of exercise, stratified by exercise type. (**d**) Mean standardised CGM traces 24 h post-exercise expressed as the deviation from the mean sedentary CGM value (zero-line). (**e**) Distribution of hourly glycaemic decrease from users’ mean sedentary value over 24 h after exercise, calculated as the mean deviation from the zero-line observed in (**d**). (**f**) Mean changes in glycaemic ranges 24 h after exercise compared with sedentary days. (**a**, **d**) Shaded ribbons indicate ±1 SEM. (**b**, **c**, **e**) Boxplots show the median (line), IQR (box) and whiskers extending to 1.5×IQR. Outliers are shown as data points. ***p<0.001

The mean CGM traces before and immediately after exercise onset, categorised by exercise type, revealed that all exercise types caused a decrease of standardised CGM value within 1 h of commencing exercise. The magnitude of the decreases differed between exercise types and varying starting values (one-way ANOVA that all exercise types start at the same value, *F*=50.33, *p*<0.001; one-sample *t* tests that starting value *z*=0, *p*<0.001 for WALK and AER, *p*=0.29 for ANAER) (Fig. 1a, b).

Furthermore, the three exercise types modulated CGM values differently (one-way ANOVA on user-level mean change values across exercise types, *F*=312.33, *p*<0.001). Exercise was noted to cause a mean decrease in CGM values of approximately (mean ± SD) −1.06±0.89 mmol/l regardless of exercise type; specifically −1.24±0.81 mmol/l for WALK, −1.43±1.02 mmol/l for AER and −0.52±0.81 mmol/l for ANAER; these decreases were all significantly different from zero (Bonferroni-corrected one-sample *t* tests of glycaemic change from onset to end of exercise against zero across exercise types; all *p*<0.001) and significantly different from each other (Tukey’s HSD; *p*<0.001 for all contrasts).

Additionally, we found that different types of exercise showed different 1 min rates of change in glucose (one-way ANOVA on user-level mean rate of change across exercise types, *F*=313.38, *p*<0.001). The exercise types showed different rates of change (per 1 min) in glucose, specifically (mean ± SD) −0.03±0.02 mmol/l for WALK, −0.04±0.03 mmol/l for AER and −0.01±0.02 mmol/l (Tukey’s HSD; *p*<0.001 for all comparisons) (Fig. 1c).

### 24 h effect of exercise on glycaemia

The 24 h post-exercise glucose response remained below the sedentary glycaemic mean (represented as the zero-line in Fig. 1d), suggesting a prolonged glucose-lowering effect of exercise on glycaemia. The mean difference from sedentary CGM values across 24 h after exercise was (mean ± SD) −0.22±0.5 mmol/l for WALK, −0.35±0.5 mmol/l for AER and −0.44±0.59 mmol/l for ANAER (Fig. 1e). This glucose-lowering effect differed between exercise types (one-way ANOVA on mean difference from the sedentary mean between exercise types, *F*=75.54, *p*<0.001); WALK showed a smaller decrease than both AER and ANAER (Tukey’s HSD; *p*<0.001 for both comparisons) and ANAER had a larger glucose-lowering effect than AER exercise (Tukey’s HSD; *p*<0.001) (Fig. 1d).

All exercise types modulated changes in all glycaemic ranges compared with sedentary data (Bonferroni-corrected one-sample *t* test against zero, *p*<0.001 for all comparisons). Additionally, the different exercise types modulated time spent in glycaemic ranges differently (one-way ANOVAs for TBR, TIR and TAR across exercise types, all *p*<0.001). Specifically, all exercise types differed in their resulting changes in proportions of TBR, TIR and TAR (*p*<0.001), except for the change of TBR between AER and ANAER activities, which increased TBR roughly equally (*p*=0.69). WALK increased TBR by 0.37±1.57%, increased TIR by 2.08±6.06% and decreased TAR by −2.45±6.24%. AER increased TBR by 0.74±1.70%, increased TIR by 2.94±6.46% and decreased TAR by −3.68±6.65%. ANAER increased TBR by 0.68±1.79%, increased TIR by 3.93±7.16% and decreased TAR by −4.61±7.49% (Fig. 1f).

### Post-exercise hypoglycaemia risk

**Fig. 2:**
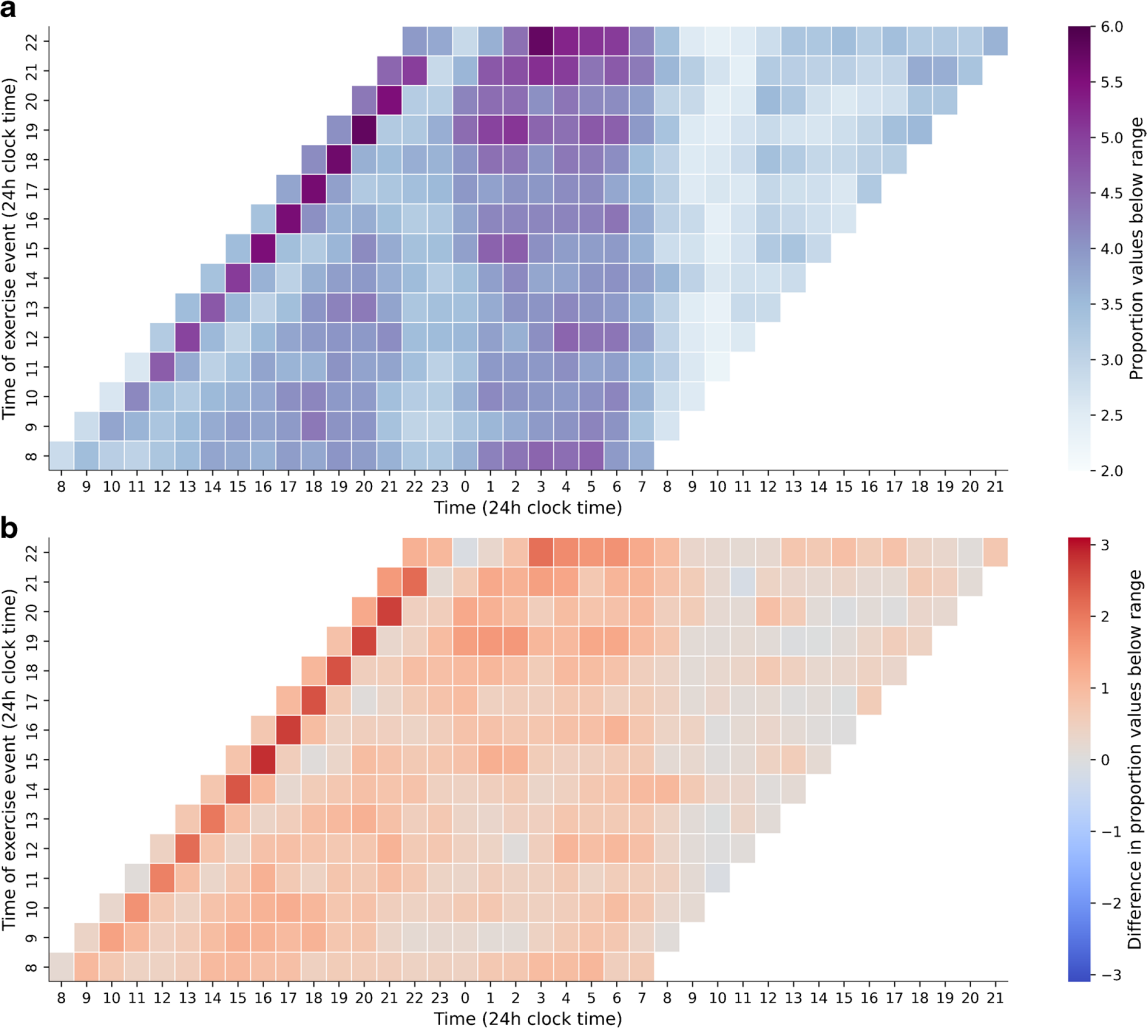
Heatmaps of mean hourly CGM proportions below range (<3.9 mmol/l) over 24 h after performing any exercise. (**a**) Absolute mean hourly CGM proportions below range 24 h after exercise. (**b**) Differences in mean hourly CGM proportions below range 24 h after exercise compared with baseline proportions on sedentary days

The mean hourly CGM proportions below range (<3.9 mmol/l glucose) were assessed 24 h after exercise onset and compared with the sedentary days, showing a significant increase, particularly 1 h after exercise onset (Fig. 2). In absolute proportions, an increase was observed throughout the nocturnal period (Fig. 2a), while in adjusted proportions, an increase was evident around the clock (Fig. 2b).

**Fig. 3:**
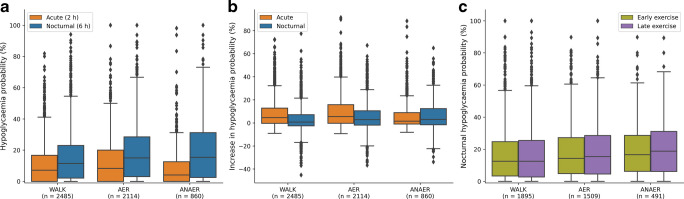
Probabilities of hypoglycaemic events across different times. (**a**) Distribution of probability of a hypoglycaemic event in the acute- (within 2 h after exercise onset) and nocturnal (00:00 hours to 06:00 hours) periods after exercise. (**b**) Increases in probability of hypoglycaemia in the acute and nocturnal periods compared with sedentary days. (**c**) Differences in nocturnal hypoglycaemia probabilities depending on early vs late exercise onset. Boxplots show the median (line), IQR (box) and whiskers extending to 1.5×IQR. Outliers are shown as points

To study the interaction between exercise type and hypoglycaemia probability at different times, acute (2 h after exercise onset) and nocturnal (00:00 hours to 06:00 hours, following exercise) hypoglycaemia times were contrasted across exercise types (Fig. 3a). For acute hypoglycaemia, a one-way ANOVA test indicated a main effect of exercise type (*F*=33.54, *p*<0.001). Tukey's HSD pairwise comparisons with family-wise error rate (FWER)=0.01 suggested that all exercise types differed, with AER yielding the largest absolute probability, followed by WALK and ANAER exercise. Similarly, for absolute nocturnal hypoglycaemia probability, a main effect of exercise type was observed (*F*=25.27, *p*<0.001). Tukey's HSD pairwise comparisons (FWER=0.01) suggested that WALK had a smaller absolute probability of nocturnal hypoglycaemia than AER and ANAER, which did not differ significantly from each other. For all exercise types, absolute hypoglycaemia was significantly more likely during the 6 h nocturnal period than the acute 2 h post-exercise period (Bonferroni-corrected *p*<0.01 for all comparisons).

Further, to assess how exercise modulates the probabilities of hypoglycaemic events compared with baseline, adjusted hypoglycaemia probabilities were calculated by subtracting baseline hypoglycaemia probabilities from the observed sedentary probabilities. For the adjusted acute probability of hypoglycaemia, a one-way ANOVA test indicated a main effect of exercise type (*F*=36.23, *p*<0.001) (Fig. 3b). Tukey’s HSD pairwise comparisons (FWER=0.01) suggest that all exercise types differed, with AER yielding the largest adjusted probability, followed by WALK and ANAER exercise. A main effect of exercise type was also found for adjusted nocturnal hypoglycaemia probability (*F*=35.19, *p*<0.001). Here, Tukey's HSD suggested that WALK had a smaller adjusted nocturnal probability than AER and ANAER, which did not differ significantly from each other. WALK and ANAER exercise showed differing adjusted hypoglycaemia probabilities between acute and nocturnal periods (Bonferroni-corrected *p*<0.01 for both); however, the contribution of ANAER to adjusted acute vs nocturnal hypoglycaemia probabilities was approximately equal (Bonferroni-corrected *p*=0.14).

Lastly, to assess whether early and late exercise timings (before or after 15:30 hours) yield a different chance of nocturnal hypoglycaemia, matched samples of 1895, 1509 and 491 users for WALK, AER and ANAER exercise, respectively, with three or more exercises in each time bin were compared in their subsequent nocturnal hypoglycaemia probabilities. Bonferroni-corrected paired-samples *t* tests suggested that exercise done in the later part of the day did not have a significantly higher probability of nocturnal hypoglycaemia than exercises done earlier in the day (Δ=0.60±0.27%, 0.80±0.29% and 1.32±0.46%; Bonferroni-corrected *p*=0.079, 0.019 and 0.013; Cohen’s *D*=0.036, 0.049 and 0.079 for WALK, AER and ANAER, respectively) (Fig. 3c). Analysis combining all exercise types to compare nocturnal hypoglycaemia risk between early and late sessions yielded a statistically significant difference. However, the extremely small magnitude of this difference (see electronic supplementary material [ESM] Fig. 1) suggests that the finding has a very small effect size and minimal practical significance, leading us to maintain separate comparisons by exercise type given that different mechanisms may influence nocturnal effects.

## Discussion

This study aimed to explore the effects of different exercise types on CGM-derived glycaemia and hypoglycaemia risk in individuals with type 1 diabetes, leveraging RWD obtained from CGM devices and Apple Health from the users of the mySugr app. Our results provide comprehensive insights into how various forms of exercise, namely WALK, AER and ANAER exercise, affect acute and extended glucose levels, as well as the associated risk of hypoglycaemia.

### Acute post-exercise glycaemia

The rising CGM slopes 1 h before all exercise types likely suggest that users exercised in a fed state or consumed carbohydrates proactively to prevent hypoglycaemia. Notably, the decreases began slightly before exercise onset, possibly due to the resampling of CGM data (contributing to short-term noise as seen for AER exercise forms), delayed manual logging or delayed automatic detection of fitness trackers during ANAER exercise, or users doing warm-ups prior to commencing ANAER training.

AER exercise induced a more-intense glycaemic decrease than ANAER exercise (Fig. 1a), resulting in significantly larger decreases from onset to end (Fig. 1b), and steeper 1 min rates of change (Fig. 1c). These findings align with the growing understanding that acute effects of ANAER exercise are less specific than those of AER exercise, which facilitate a larger acute decrease of CGM values.

Notably, WALK induced a smaller decrease than more-intense AER, likely due to its lower intensity. Nonetheless, WALK substantially reduced glycaemia, suggesting an immediate benefit for glucose management.

Importantly, the distributions observed in Fig. 1b are mean within-user changes, not individual event changes. This implies the directionality of changes was relatively consistent within each user, and individual differences in response to exercise type likely existed. Further RWD studies could separate exercise effects by estimated insulin- and carbohydrate-on-board levels from users’ logs to explore how insulin levels and prandial states influence glycaemic concentrations. The answer to the latter question could potentially shed further light on interpreting over three decades of findings from experimental research [25, 26].

### Extended post-exercise glycaemia

All exercise types reduced glucose levels over 24 h and potentially longer, with CGM values consistently below the sedentary mean (Fig. 1d, e) and decreased TAR in favour of TIR and TBR compared with sedentary data (Fig. 1f). The magnitudes of glycaemic decreases differed across exercise types, with the most pronounced effects observed for ANAER exercise. Despite a smaller acute impact, ANAER provided sustained, long-term glucose suppression, possibly more evenly distributed throughout the day than AER exercise, based on CGM traces. Notably, while ANAER and AER exercise resulted in similar TBR increases, ANAER led to a significantly greater TIR increase, suggesting a superior relative benefit by improving TIR at a comparable TBR risk.

### Post-exercise hypoglycaemia

Our research confirmed a biphasic post-exercise hypoglycaemia risk, notably in the hour after exercise onset (seen in Fig. 2a as ‘the diagonal’ across the 1 h offset from exercise onset), and during nocturnal hours. Beyond these periods, exercise also appears to elevate baseline hypoglycaemia risk around the clock (Fig. 2b). Nocturnal hypoglycaemia risk is considerable even without exercise but is further exacerbated by it (Fig. 2a, b). In absolute terms, post-exercise hypoglycaemia was more likely during the 6 h nocturnal period than the 2 h acute window (Fig. 3a), at least partly due to the longer observation time in the nocturnal period. Interestingly, while all exercise types contributed to both acute and nocturnal risk of hypoglycaemia, AER forms of exercise (including WALK) increased the acute risk more than the nocturnal risk. In contrast, ANAER's contribution was similar for both periods (Fig. 3b), suggesting that its metabolic effects, by preventing sharp glucose decreases, also reduce acute hypoglycaemia probability.

Finally, we assessed whether early (before 15:30 hours) vs late exercise timing affected nocturnal hypoglycaemia probability. Although the probabilities of nocturnal hypoglycaemia were higher for exercise sessions done after 15:30 hours, the *p* values above the 0.01 threshold, and the small effect size, suggest that nocturnal hypoglycaemia was not significantly different depending on the timing of the exercise during the day (Fig. 3c). Instead, it seems that the increase in nocturnal hypoglycaemia risk is uniform regardless of when exercise is performed, further supporting the sustained 24 h glucose-lowering effect of exercise. This contrasts with studies linking later exercise to higher nocturnal hypoglycaemia risk [13, 16], perhaps partly because our highly active sample was skilled in overcoming post-exercise hypoglycaemia (Fig. 3c). When collapsing all exercise types, the comparison of early vs late exercise timing showed a statistically significant difference in nocturnal hypoglycaemia probability, although the effect size was very small, suggesting that the difference may not be clinically relevant (ESM Fig. 1). Further studies are needed to clarify the role of exercise timing.

### Strengths, limitations and further directions

This RWD study uses extensive exercise data covered by CGM to derive generalised insights into how different types of exercise impact glycaemia. It does not fully account for all variables influencing glycaemia around exercise times, including active carbohydrate and insulin concentrations, insulin delivery modalities, exercise intensities and other individual- and exercise-related factors. The study did not separate events by originating exercise-tracking or CGM devices, opting for an inclusive and device-agnostic analysis. Similarly, the effects of sex on the observed glycaemic and hypoglycaemia outcomes were not explored, with the focus placed on deriving population-wide insights. The extensive dataset of nearly half a million exercise events from 3248 users allows for robust observations from real-world settings and offers high generalisability. Glycaemic comparisons between exercise types are based on different (but somewhat overlapping) subsamples, which may not fully account for individual effects of different exercise types. However, as the subsamples mostly included individuals who regularly performed specific activity types, it may support findings specific to individuals in those habit-groups. The analysis focused exclusively on the main effects of individual exercise sessions, thus the cumulative glycaemic impact of consecutive or repeated exercise events as well as modelling of duration on 24 h post-exercise glucose levels remain unexplored within the scope of the present work.

We acknowledge the potential for unlogged physical activity to contaminate sedentary-day comparisons, though selecting for users who consistently log exercise helps mitigate this inherent limitation of RWD acquisition. Furthermore, even highly objective studies, such as the T1DEXI papers, relied on participants manually self-reporting their exercise sessions, and analysing comprehensive accelerometer data for a cohort of this magnitude remains a significant practical challenge.

It is foreseeable that data from commercially available wearable sensors will become more widespread, driven by hardware innovations of CGM technology and other wearable concepts capable of collecting reliable high-frequency biomarker data. Notably, initiatives such as the T1DEXI (and T1DEXI-P), as well as the recent Apple Heart and Movement Study from Apple and Brigham and Women's Hospital, which involves the collection of sensor data from the Apple Watch, as well as patient-reported outcome (PRO) from participants [27], show the increasing scale of real-world biomarker data studies, thereby offering new possible directions for study.

The development of user-centred (diabetes management) solutions that collect large amounts of data presents an opportunity for further refining pseudo-experimental real-world study designs. This approach offers the advantage of having years of individual user data, allowing for per-patient understanding of variations in glycaemic patterns, response to interventions and lifestyle impacts. This paradigm offers a novel way to conduct research, complementing findings from experimentally controlled laboratory trials, which traditionally do not collect comparable amounts of biomarker data.

Ultimately, integrating advanced wearable technologies and RWD analytics will pave the way for more personalised and effective diabetes management strategies.

This RWD study provides valuable insights for individuals with type 1 diabetes, helping them gain a deeper understanding of how different types of exercise influence glycaemic management and hypoglycaemia risk. These findings can assist healthcare professionals in offering personalised recommendations for effective daily diabetes management, aiming to enhance quality of life. Finally, these developments will drive the creation of algorithms designed to assist patients in managing their diabetes by predicting glycaemic responses to various forms of exercise, enabling more precise and timely interventions. Taking this together, our RWD findings holistically confirm the robustness and significance of previous research.

## Supplementary Information

Below is the link to the electronic supplementary material.

ESM Figure (PDF 175 KB)

## Data Availability

Anonymised data, without any demographic identifiers, underlying the results and analysis can be made available to researchers upon reasonable request to the corresponding author after publication. A data access agreement needs to be signed in advance.

## Abbreviations

AER: Aerobic, excluding walking (exercise category)
ANAER: Anaerobic (exercise category)
CGM: Continuous glucose monitoring
FWER: Family-wise error rate
HSD: Honestly significant difference
RWD: Real-world data
TAR: Time above range
TBR: Time below range
TIR: Time in range
WALK: Walking (exercise category)
