# Supplementary material for: Exercise in type 1 diabetes: real-world data on glucose levels and hypoglycaemia risk from over 420,000 exercise sessions
Source: Diabetologia. 2026 Feb 13;69(6):1457–67. doi: 10.1007/s00125-026-06672-y (PMC13109116; doi:10.1007/s00125-026-06672-y)
Supplement: Supplementary file 1 — ESM Figure (PDF 175 KB) [file 125_2026_6672_MOESM1_ESM.pdf]

Supplementary figures

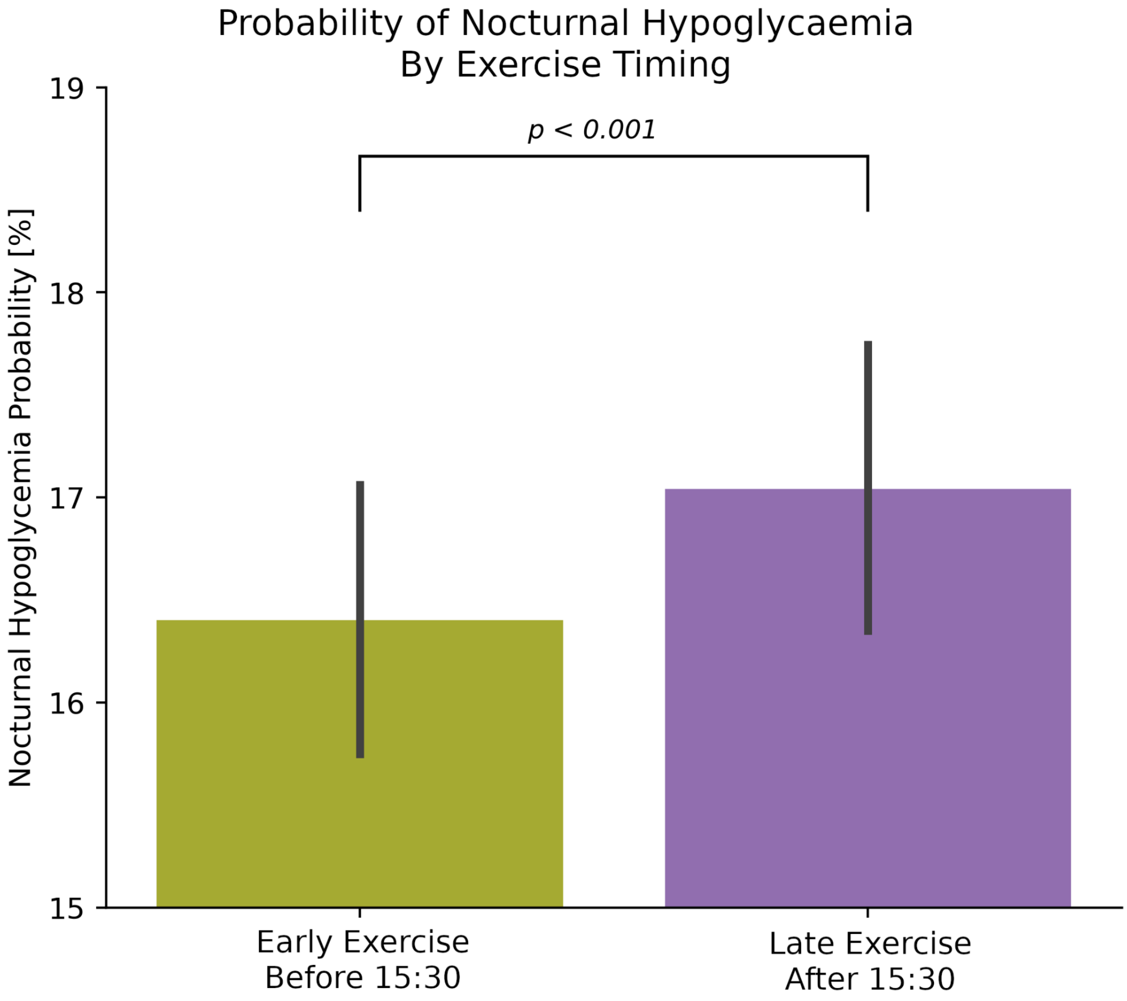

ESM Fig. 1 - Probabilities of hypoglycemic events across different times. Barplots show the mean nocturnal hypoglycemia probability in % (error bars reporting the 95% confidence interval), stratified by early and late exercise onset. Differences were tested using a paired, two-sided t-test.
